# Exogenous Spermidine Promotes γ-Aminobutyric Acid Accumulation and Alleviates the Negative Effect of NaCl Stress in Germinating Soybean (*Glycine max* L.)

**DOI:** 10.3390/foods9030267

**Published:** 2020-03-02

**Authors:** Weiming Fang, Fei Qi, Yongqi Yin, Zhengfei Yang

**Affiliations:** College of Food Science and Engineering, Yangzhou University, Yangzhou, Jiangsu 210095, China; wmfang@yzu.edu.cn (W.F.);

**Keywords:** germinating soybeans, γ-aminobutyric acid, spermidine, NaCl stress

## Abstract

We investigated the effects of exogenous spermidine (Spd) on the physiological status, γ-aminobutyric acid (GABA) synthase activity, and gene expressions in germinating soybeans under NaCl stress. The results show that Spd significantly increases sprout growth and biomass, decreases malonaldehyde and H_2_O_2_ contents, and markedly promotes the activities of superoxide dismutase, catalase, peroxidase, and glutathione peroxidase of germinating soybeans. The harmful effect of NaCl stress was alleviated by exogenous Spd. GABA accumulation in germinating soybeans was caused by the activation of diamine oxidase, polyamine oxidase, aminoaldehyde dehydrogenase, and glutamate decarboxylase activities and by up-regulating their gene expression under Spd-NaCl treatment. The GABA content decreased by 57% and 46% in germinating soybeans with the application of aminoguanidine under Spd and Spd-NaCl treatments, respectively. In conclusion, spermidine induces the accumulation of GABA and increases sprouts biomass, thereby enhancing the functional quality of germinating soybeans.

## 1. Introduction

Gamma-aminobutyric acid (GABA), a four-carbon non-protein amino acid, is a major inhibitory neurotransmitter in the mammalian brain [1]. Clinical research showed that it can lower blood pressure, prevent cancers, and reduce cardiovascular diseases [2,3,4]. However, the GABA content in natural food is far from meeting the physiological needs of the human body. Hence, many functional foods and dietary supplements with GABA as the main raw material are supplied on the market [5,6]. Currently, the most common and effective methods to accumulate GABA in edible plants include germinating under stressful conditions such as salt stress. However, the concomitant growth and biomass of germinating plants are markedly suppressed [7,8,9].

Polyamines (PAs) are commonly known to have protective effects on abiotic stress. The application of exogenous PAs can improve the inhibition of growth, increase the level of endogenous PAs, remove reactive oxygen species (ROS), protect the activity of the enzyme system, and maintain the integrity of the cell function of plants under abiotic stress [10,11]. More importantly, in higher plants, PAs is the substrate of GABA. GABA can be formed through the polyamine degradation pathway where polyamine is firstly converted to γ-aminobutyraldehyde by diamine oxidase (DAO, EC 1.4.3.6) or polyamine oxidase (PAO, EC 1.5.3.11) and then γ-aminobutyraldehyde is converted to GABA by aminoaldehyde dehydrogenase (AMADH, EC1.2.1.19). Meanwhile, it can be synthesized by glutamate decarboxylation via glutamate decarboxylase (GAD, EC 4.1.1.15), which is called GABA shunt [12]. Studies demonstrated that exogenous application of PAs can increase phytochemicals in various plants such as mung bean and cucumber seedlings [11,13,14,15]. These studies indicate that PAs might be an ideal biotechnological target for improvement of GABA-enriched plant grown under stressful conditions.

Soybean (*Glycine max* L.) is one of the most important foods due to its nutritional and pharmacological importance. GABA-enriched germinating soybeans can be harvested under NaCl stress [8,16,17]. However, soybean is a salt-sensitive crop, as NaCl stress seriously inhibits its growth and reduces its biological yield [16]. According to our previous studies, spermidine (Spd), one of the important PAs, was selected as the most appropriate agent and its concentration was optimized. Exogenous Spd (0.10 mM) countered the harmful effects of NaCl (50 mM) stress and increased the biomass and GABA content in germinating soybeans. However, the precise molecular mechanism underlying the role of Spd in resistance to NaCl stress is not yet completely understood.

In the present study, the effect of Spd treatment on the physiology, activities, and gene expressions of GABA key synthases (including DAO, PAO, AMADH, and GAD) in germinating soybean under NaCl stress were investigated. The objective was to reveal the molecular mechanism of Spd on GABA accumulation in soybeans under NaCl stress during germination.

## 2. Materials and Methods

### 2.1. Materials and Reagents

Soybean seeds (cv. Yunhe) were purchased from Jilin Academy of Agricultural Sciences (Jilin, China) in 2017 and stored at −18 °C prior to use. Spermidine (purity >99%), GABA standard, p-dimethylaminobenzene sulfonyl chloride, and aminoguanidine (AG, specific inhibitor of DAO) were purchased from Sigma-Aldrich (Shanghai, China). A plant RNA extraction kit (R6827) was purchased from Omega (GA, USA); and the RNA reverse transcription kit (RR047A), TB Green Premix Dimer Eraser (RR091A), and ROX Reference Dye (RR091A) were purchased from Takara (Dalian, China). All other reagents were analytical grade.

### 2.2. Material Treatment and Experimental Design

#### 2.2.1. Effects of Exogenous Spd on Physiology and Biochemistry of Germinating Soybeans under NaCl Stress

The dry seeds were washed with deionized water and soaked in 1% (*v*/*v*) NaClO solution for 15 min. After disinfection, they were steeped with distilled water at 30 ± 1 °C for 4 h. The soaked seeds were then placed in a bean sprouting machine to germinate in a dark incubator at 30 ± 1 °C with water containing the following additives: (1) Control: deionized water; (2) NaCl stress treatment: 50 mM NaCl; (3) Spd treatment: 0.10 mM Spd; and (4) NaCl + Spd treatment: 50 mM NaCl + 0.10 mM Spd. The culture solution was replaced every 1 day. Germinating soybeans were randomly sampled on the 4th and 6th day and frozen in liquid nitrogen for further biochemical measurements.

#### 2.2.2. Effect of Exogenous Spd on GABA Metabolism of Germinating Soybeans under NaCl Stress

Dry seeds were sterilized, steeped, and germinated as stated above. The treatments were as follows: Control: deionized water; (2) NaCl stress treatment: 50 mM NaCl; (3) Spd treatment: 0.10 mM Spd; (4) NaCl + Spd treatment: 50 mM NaCl + 0.10 mM Spd; (5) NaCl + AG treatment: 50 mM NaCl + 1.50 mM AG; (6) NaCl + Spd + AG treatment: 50 mM NaCl + 0.10 mM Spd + 1.50 mM AG; and (7) Spd + AG treatment: 0.10 mM Spd + 1.50 mM AG. AG is a specific inhibitor of DAO, blocking the polyamine degradation pathway. Our previous experiment showed that 5th day germinating soybeans under AG treatment were decayed and softened, which could not be sampled for further study. Hence, germinating soybeans were randomly sampled on the 2nd and 4th day for further biochemical measurements.

### 2.3. Measurement of Main Physiological and Biochemical Indexes

Sprout length and respiratory rate were measured as described by Yin et al. [18]. Malondialdehyde (MDA) content was measured following the method reported by Madhava and Sresty [19]. Contents of H_2_O_2_, soluble protein, soluble sugar, and free amino acids were measured according to the method reported by Yin et al. [16].

### 2.4. Measurement of Antioxidant Activities

The superoxide dismutase (SOD), catalase (CAT), peroxidase (POD), and glutathione peroxidase (GPX) activities were determined using a SOD Assay Kit (A001-1 Nanjing Jiancheng Bioengineering Institute, Nanjing, China), CAT Assay Kit (A007-1 Nanjing Jiancheng Bioengineering Institute, Nanjing, China), POD Assay Kit (A084-3 Nanjing Jiancheng Bioengineering Institute, Nanjing, China), and GPX Assay Kit (A004-3 Nanjing Jiancheng Bioengineering Institute, Nanjing, China), respectively, according to the manufacturer’s instructions.

### 2.5. Measurements of GABA Contents

GABA content was analyzed as described by Yang et al. [20].

### 2.6. GAD, DAO, PAO, and AMADH Activity Assay

GAD, DAO, and AMADH activity were determined according to Yin et al. [18]. PAO activity was determined according to Yang et al. [20].

### 2.7. GABA Synthase Gene Expression (Quantitative Real-Time PCR).

Total RNA was isolated from mustard sprouts using an E.Z.N.A™ Plant RNA Kit (R6827-01, Omega, GA, USA). The RNA samples were reverse transcribed into cDNA. Triplicate quantitative assays were performed on each cDNA using SYBRR Premix Ex-Taq™ (RR420A, Takara, Dalian, China) and an ABI 7500 sequence detection system (Applied Biosystems, Foster City, CA, USA) according to the manufacturer’s protocol. The sequence-specific primers that were used in this study are shown in Table 1. 

### 2.8. Statistical Analyses

All experiments in this study were repeated at least three times in independent experiments. Average values and standard deviations were computed according to the experimental data. One-way analysis of variance (ANOVA) with Tukey’s test were conducted on the data and a *p*-value of 0.05 was considered significant. The gene relative expression was analyzed using the 2^−ΔΔCt^ method [21].

## 3. Results

### 3.1. Growth Characteristics of Germinating Soybeans

**Figure 1:**
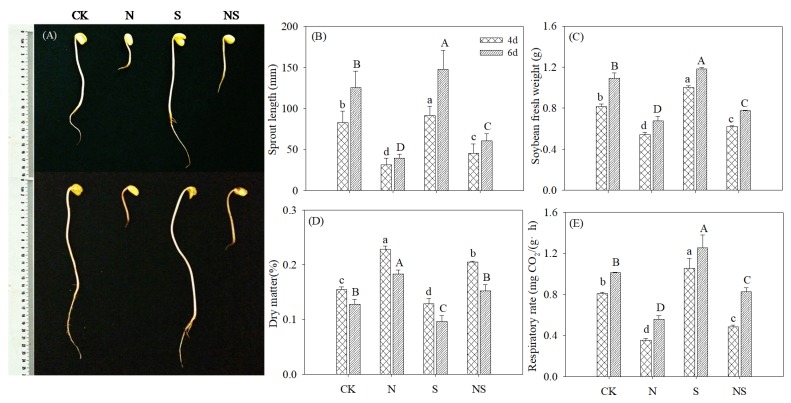
Changing patterns in a (**A**) photograph in terms of (**B**) sprout length, (**C**) fresh weight, (**D**) dry matter content, and (**E**) respiratory rate in germinating soybeans. Lowercase letters reflect the significance of differences in index among treatments of 4-day germinating soybeans and capital letters reflect the significance of differences in index among treatments of 6-day germinating soybeans (*p* < 0.05). CK, Control; N, NaCl; S, Spd; NS, NaCl + Spd.

As shown in Figure 1A, compared with the control, NaCl treatment significantly inhibited the growth and development of germinating soybeans, whereas the addition of Spd alone significantly promoted growth and the combination of Spd with NaCl treatment effectively alleviated the growth inhibition caused by stress during germination (Figure 1A). Similarly, a stimulatory effect of Spd under NaCl stress was also observed on the sprout length, fresh weight, and respiration rate in germinating soybeans compared with the NaCl treatment (Figure 1B–E).

### 3.2. Soluble Protein, Soluble Sugar, MDA, and H_2_O_2_ Contents in Germinating Soybeans

**Figure 2:**
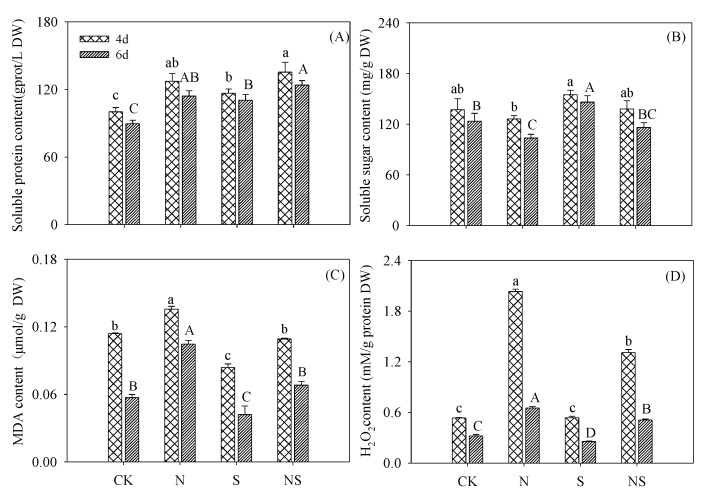
Changing pattern of (**A**) soluble protein content, (**B**) soluble sugar content, (**C**) malonaldehyde (MDA) content, and (**D**) H_2_O_2_ content in germinating soybeans. Lowercase letters reflect the significance of differences in index among treatments of 4-day germinating soybeans and capital letters reflect the significance of differences in index among treatments of 6-day germinating soybeans (*p* < 0.05). CK, Control; N, NaCl; S, Spd; NS, NaCl + Spd.

Compared with the control, the soluble protein content in germinating soybeans increased significantly under other treatments during germination (*p* < 0.05; Figure 2A). The difference in soluble sugar content under Spd-NaCl treatment was not statistically significant compared with NaCl treatment (Figure 2B). During germination, compared with NaCl treatment, MDA and H_2_O_2_ contents both decreased significantly in the control and following treatment with Spd (*p* < 0.05) (Figure 2C,D). We found no significant difference in MDA content between the control and Spd-NaCl treatment (*p* > 0.05). With the combined Spd with NaCl treatment, H_2_O_2_ content was significantly higher than that of the control and Spd treatment.

### 3.3. Antioxidant Enzyme Activity

**Figure 3:**
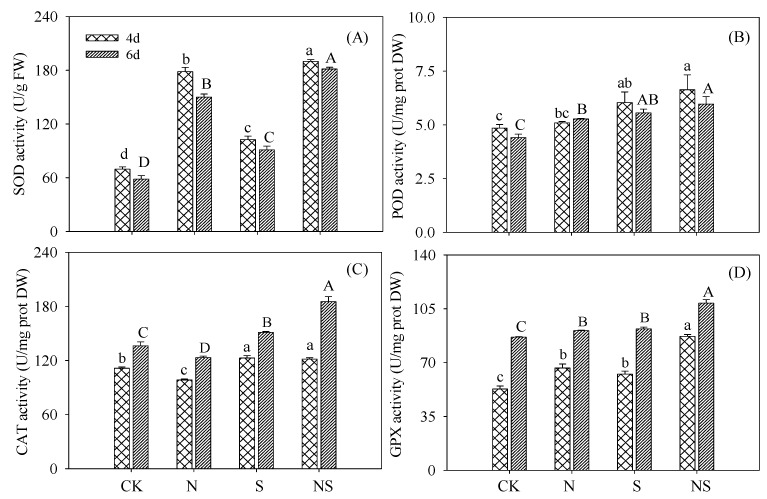
Changes in (**A**) superoxide dismutase (SOD), (**B**) peroxidase (POD), (**C**) catalase (CAT), and (**D**) glutathione peroxidase (GPX) activity in germinating soybeans. Lowercase letters reflect the significance of differences in index among treatments of 4-day germinating soybeans and capital letters reflect the significance of differences in index among treatments of 6-day germinating soybeans (*p* < 0.05). CK, Control; N, NaCl; S, Spd; NS, NaCl + Spd.

Compared with the control and the addition of spermidine alone, both SOD and CAT activities changed significantly under NaCl treatment (*p* < 0.05) (Figure 3A,C). The POD and GPX activities in germinating soybeans treated with NaCl were not significantly (*p* > 0.05) different compared with the soybeans under Spd treatment, whereas they were significantly (*p* < 0.05) higher than in those under the control (Figure 3B,D). Under Spd-NaCl treatment, the activities of major antioxidant enzymes (SOD, POD, CAT, and GPX) increased significantly (*p* < 0.05) in germinating soybeans during germination compared with NaCl treatment (Figure 3).

### 3.4. GABA Content in Germinating Soybeans

**Figure 4:**
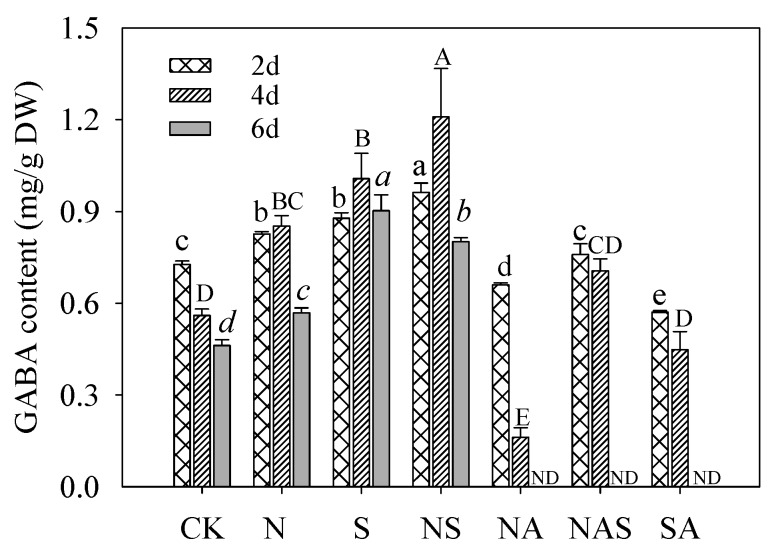
Effect of Spd on γ-aminobutyric acid (GABA) content in germinating soybeans. Lowercase letters reflect the significance of differences in index among treatments of 2-day germinating soybeans, capital letters reflect the significance of differences of 4-day germinating soybeans, and lowercase letters in italics reflect the significance of differences of 6-day germinating soybeans (*p* < 0.05). CK, Control; N, NaCl; S, Spd; NS, NaCl + Spd; NA, NaCl + AG; NAS, NaCl + AG + Spd; SA, Spd + AG; AG, Aminoguanidine; ND, Non detection.

After germinating for four days and six days under NaCl treatment, the GABA content increased significantly (*p* < 0.05), being 1.52- and 1.32-fold higher than the control in germinating soybeans, respectively (Figure 4). Compared with the control and NaCl treatment, the GABA content in four-day germinating soybeans under Spd-NaCl treatment increased by 116% and 73% compared with the control and increased by 42% and 74% compared with the NaCl treatment, respectively (Figure 4). Moreover, compared with the Spd treatment, when AG was added under the Spd treatment, the GABA content in two-day and four-day germinating soybeans decreased by 35% and 58%, respectively. The addition of AG resulted in a decrease in GABA content by 21% and 46% in two-day and four-day germinating soybeans under Spd-NaCl treatment, respectively (Figure 4). The content of GABA synthesized by the polyamine degradation pathway under Spd and NaCl-Spd treatments accounted for 57% and 46% of the total GABA content in four-day germinating soybeans, respectively.

### 3.5. Changes in GABA Synthase Activity

**Figure 5:**
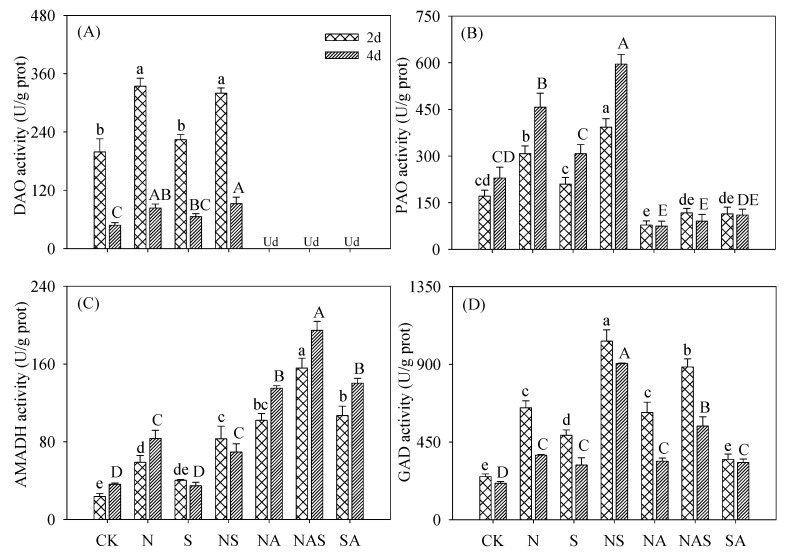
Changes in (**A**) diamine oxidase (DAO), (**B**) polyamine oxidase (PAO), (**C**) aminoaldehyde dehydrogenase (AMADH), and (**D**) glutamate decarboxylase (GAD) activity in germinating soybeans. Lowercase letters reflect the significance of differences in index among treatments of 2-day germinating soybeans and capital letters reflect the significance of differences in index among treatments of 4-day germinating soybeans (*p* < 0.05). CK, Control; N, NaCl; S, Spd; NS, NaCl + Spd; NA, NaCl + AG; NAS, NaCl + AG + Spd; SA, Spd + AG; AG, Aminoguanidine; Ud, Undetected.

DAO activity decreased with germination time and could not be detected after AG addition. After germinating for two days under Spd-NaCl treatment, DAO activity was 2.65- and 1.64-fold higher than the control and the NaCl treatment, respectively (Figure 5A). The presence of AG also decreased PAO activity in germinating soybeans. Spd-NaCl treatment dramatically increased PAO activity during germination compared with the other treatments (Figure 5B). AMADH activity in germinating soybeans under both NaCl and Spd-NaCl treatments increased significantly (*p* < 0.05) compared with the control and Spd treatment. The addition of AG increased AMADH activity during germination (Figure 5C). Compared with other treatments, GAD activity under Spd-NaCl treatment also increased markedly during germination (Figure 5D). The presence of AG significantly decreased GAD activity under Spd-NaCl treatment, whereas we found no significant difference (*p* > 0.05) under NaCl treatment.

### 3.6. Changes in GABA Synthase Gene Expression

#### 3.6.1. GADs Relative Expression

**Figure 6:**
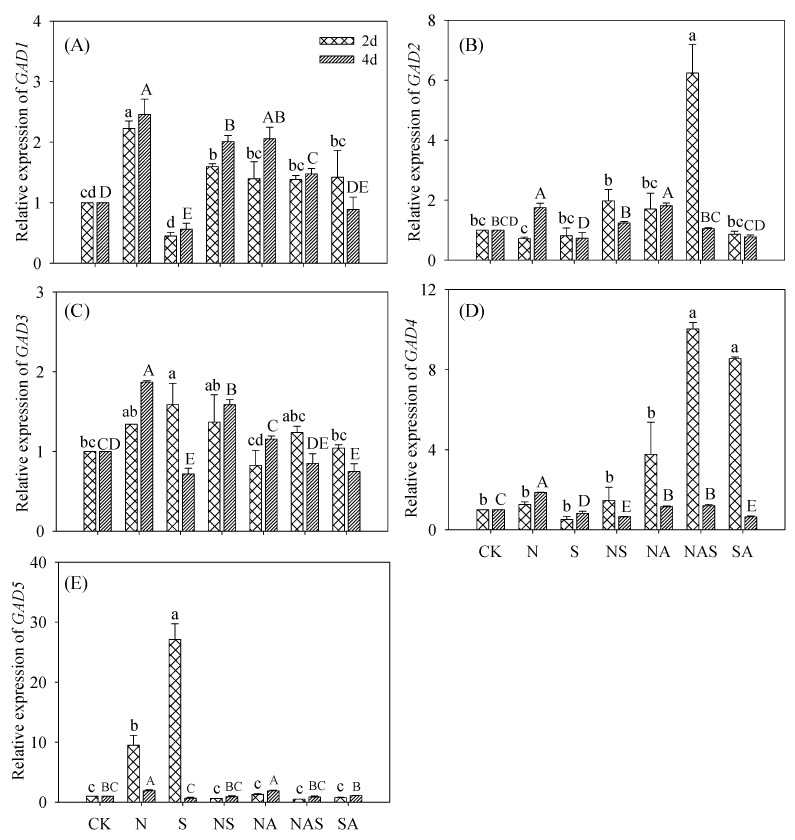
Changes of (**A**) *GAD1*, (**B**) *GAD2*, (**C**) GAD3, (**D**) *GAD4*, and (**E**) *GAD5* relative expression in germinating soybeans. Lowercase letters reflect the significance of differences in index among treatments of 2-day germinating soybeans and capital letters reflect the significance of differences in index among treatments of 4-day germinating soybeans (*p* < 0.05). CK, Control; N, NaCl; S, Spd; NS, NaCl + Spd; NA, NaCl + AG; NAS, NaCl + AG + Spd; SA, Spd + AG; AG, Aminoguanidine.

Compared with the control, the NaCl and Spd-NaCl treatments significantly induced *GAD1* and *GAD3* expression during germination (*p* < 0.05). Under the Spd-NaCl treatment, *GAD1* and *GAD3* expression in two-day germinating soybeans and all *GADs* expression in four-day soybeans decreased, whereas *GAD2* expression in two-day germinating soybeans increased compared with the NaCl treatment (Figure 6). The addition of AG markedly induced *GADs* expression in germinating soybeans. These results suggest that variations in the activities of GABA synthase are in accordance with gene expression.

#### 3.6.2. DAOs, PAOs, and AMADHs Relative Expression

**Figure 7:**
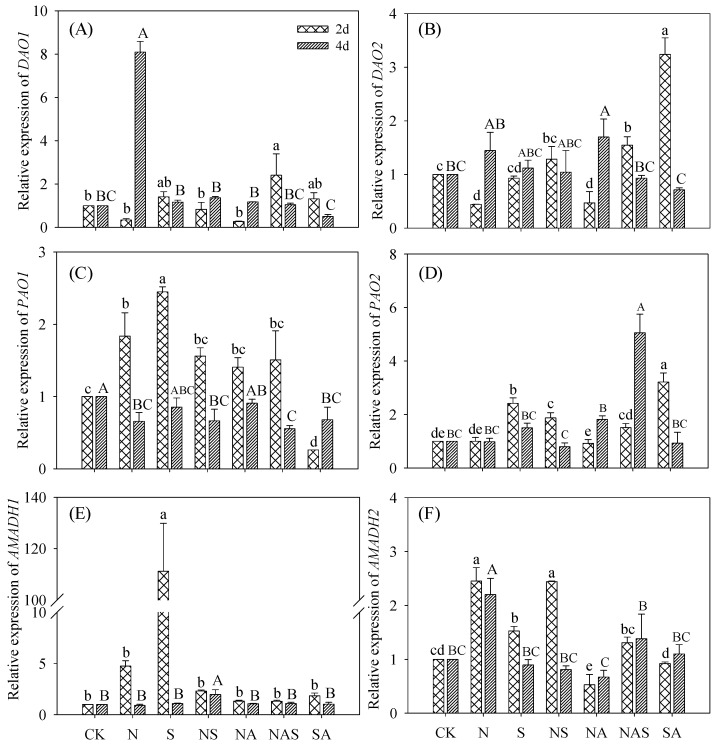
Changes of (**A**) *DAO1*, (**B**) *DAO2*, (**C**) *PAO1*, (**D**) *PAO2*, (**E**) *AMADH1*, and (**F**) *AMADH2* relative expression in germinating soybeans. Lowercase letters reflect the significance of differences in index among treatments of 2-day germinating soybeans and capital letters reflect the significance of differences in index among treatments of 4-day germinating soybeans (*p* < 0.05). CK, Control; N, NaCl; S, Spd; NS, NaCl + Spd; NA, NaCl + AG; NAS, NaCl + AG + Spd; SA, Spd + AG; AG, Aminoguanidine.

Compared with the control, the NaCl treatment significantly increased four-day *DAO1,* two-day *PAO1*, and *AMADH2* expressions, but decreased two-day *DAO1* and four-day *PAO1* expressions (*p* < 0.05), and other gene expressions were not significantly changed (Figure 7). The expressions of *DAO2*, *PAO2,* and *AMADH1* in two-day germinating soybeans increased significantly under Spd-NaCl treatment (*p* < 0.05), whereas *DAO1* and *AMADH2* in four-day germinating soybeans decreased significantly compared with the NaCl treatment. Compared with the NaCl and Spd-NaCl treatments, *DAO2 and PAO1* expressions in germinating soybeans were not significantly affected by AG during germination (*p* < 0.05; Figure 7).

## 4. Discussion

In this study, exogenous Spd effectively alleviated the growth inhibition caused by NaCl stress in germinating soybeans. Salt stress leads to water exosmosis in plants and elevated levels of ROS produce oxidative stress [22]. The growth and biological yield of germinating soybeans was inhibited by NaCl treatment (Figure 1). MDA and H_2_O_2_ contents, as indicators of membrane damage and ROS level, increased significantly (Figure 2). Spd is an internal and external stress signal molecule; its addition under NaCl stress-induced germinating soybeans initiated regulations mechanisms to alleviate the stress damage. In the present study, GABA-enriched germinating soybeans were germinated in a dark artificial environment and soybeans cannot absorb nutrients from the external environment and especially cannot synthesize organic compounds via photosynthesis. The growth of germinating soybeans under NaCl stress was inhibited, resulting in soybeans that were unable to use storage nutrients for growth during the germination stage and, therefore, showed the highest dry matter content. The activities of major ROS detoxifying enzymes (SOD, POD, CAT, and GPX) increased significantly (*p* < 0.05) in germinating soybeans under Spd-NaCl treatment. This result is consistent with a study of other soybeans [23] and salt-responsive plants [14,24,25]. MDA and H_2_O_2_ contents were decreased significantly in germinating soybeans. GABA is another of the stress-tolerance substances and functions to regulate infiltration in plants [26]. The application of Spd under the NaCl treatment significantly increased the GABA content in soybeans. These results support the conclusion that exogenous application of Spd results in salt stress resistance via the induction of antioxidant enzyme activity and GABA content, which also suggests that germinating soybeans under Spd-NaCl treatment can be used as a material to produce GABA-enriched functional ingredients.

The level of GABA in plants is mediated by GABA synthase gene expression and GABA synthase activities. In the present study, the activities of DAO, PAO, GAD, and AMADH, which are key GABA synthases, increased significantly (*p* < 0.05) under NaCl treatment compared with the controls, where the largest increase (52%) in GABA content was observed. This result is consistent with a study of other plants under abiotic stress [27,28]. GABA content increased significantly with Spd (*p* < 0.05) under NaCl treatment, and this increment was due to the increase of GAD and PAO activities. No significant change of DAO and AMADH activities in the Spd-NaCl treatment was observed. Under the Spd-NaCl treatment, expression of *DAO*2 and *PAO*2 in two-day germinated soybeans were dramatically increased while *DAO*1 in four-day germinating soybeans was strongly suppressed compared with the NaCl stress. Furthermore, five *GAD* genes (*GAD* 1 to 5) displayed differential expression either in their transcript abundance or in their expression patterns under different environmental conditions like flooding, salt, drought, and cold [29,30,31]. Compared with the control, both the NaCl and Spd-NaCl treatments significantly induced *GAD1* and *GAD3* expression during germination. These results indicate that compared with other *GADs*, *GAD1* and *GAD3* might be an important *GAD* family for salt and Spd responses in soybeans. It is noteworthy that the expression of all *GADs* in four-day germinating soybeans was lower after the addition of Spd, which is inconsistent with the increased GABA content compared with the NaCl treatment. This result suggests that the increment of GAD activity was caused by the stimulation of conditions instead of *GADs* expression. Furthermore, NaCl stress can up-regulate the expression of *AMADH2* but has no significant effect on the expression of *AMADH1*. This is consistent with the results of a study where NaCl stress was found to up-regulate the expression of rice *BADH1* in the *AMADH* gene family [32]. Although the above results indicate a correlation between GABA accumulation and the expression of soybean GABA synthase genes under Spd-NaCl treatment, the expression profiles of these genes in various tissues still require further study to fully understand this relationship.

AG is a specific inhibitor of DAO and can inhibit polyamine degradation [20]. In this study, the addition of AG completely suppressed DAO activity and then decreased GABA content. Mechanisms have been suggested for the production of GABA, the dynamic balances of GABA shunt, and polyamine degradation pathway in plants. The polyamine degradation pathway was reported to contribute 39% of GABA accumulation in soybeans under salt stress. However, in this study, the results indicated that 57% and 46% of GABA accumulation was contributed by the PA degradation under Spd and Spd-NaCl treatments, respectively. We suggest that the polyamine degradation pathway in germinating soybeans occupies an important proportion of the GABA synthesis pathway.

## 5. Conclusions

Spd significantly increased antioxidant enzyme activity and decreased MDA and H_2_O_2_ contents in germinating soybeans under NaCl stress. The activities of GABA synthase were increased significantly and the expressions of the GABA synthase gene were markedly regulated by Spd. The addition of Spd changed the contribution ratio of GABA shunt and the polyamine degradation pathway for GABA formation under NaCl stress. As a result, exogenous Spd alleviated the harmful effect of NaCl stress and increased the biomass and GABA content, which is beneficial for the enhancement of nutrition health value in germinating soybeans.

## Figures and Tables

**Table 1:** Sequence-specific primer used for quantitative real-time (qRT)-PCR assay in germinating soybean.

Primer	Sequences (5′–3′)
GAD1-F ^1^	AGCAGGTGAAGAAAATGACGA
GAD1-R ^2^	TCTTCTTCCTGTCCATCACAAA
GAD2-F	CCACTCACCCAGATGAAAAAG
GAD2-R	AGCTGCATCCTTTGGTATTGA
GAD3-F	TTCCTGTGTGAGTACGTGTGC
GAD3-R	CATCATTGCGCTCATAATCCT
GAD4-F	GGTGAGAAGATTAAGAAAGCTGC
GAD4-R	GGTAAGCCTAGCATGCTCCA
GAD5-F	CTCAGTGCAGAAGAAAATGGC
GAD5-R	ACACCCCCTTGAAGCTAACAC
DAO1-F	AGATGATACTTTAGCCGTTTGGAC
DAO1-R	CAATAAGCAGATCGCATTTCAC
DAO2-F	TCCTTGCAAGCAGTTGTGTC
DAO2-R	TGGTTTGATGGATTCCCATT
PAO2-F	GGCAGCTTTCTTGAAACACA
PAO2-R	TAACCCAAGTAGCCAAACCC
PAO2-F	CTTCCTCCAAGGAAAGCAAG
PAO2-R	GTGTCCACCTCGAGTTGTTG
AMADH1-F	TGAAGCTGGTGCTCCTTTGT
AMADH1-R	AAGATGGTCCATTCAGCAGT
AMADH2-F	TGAAGCGGGTGCTCCTTTAG
AMADH2-R	AATATGGTCCATTCAGCAGC
UBi-F	GTGTAATGTTGGATGTGTTCCC
UBi-R	ACACAATTGAGTTCAACACAAACCG

^1^ F: Forward primer sequence, ^2^ R: Reverse primer sequence.
